# Collaborative tailoring of the Reach Out, Stay Strong Essentials (ROSE) program for pregnant veterans in the U.S. Veterans Health Administration: a qualitative case study of contextual conditions and adaptations

**DOI:** 10.1186/s12913-025-12871-x

**Published:** 2025-05-23

**Authors:** Erin P. Finley, Alison B. Hamilton, Ismelda Canelo, La Shawnta S. Jackson, Rachel Lesser, Rebecca S. Oberman, Julia Yosef, Joya G. Chrystal, Erica H. Fletcher, Bevanne Bean-Mayberry, Tannaz Moin, Melissa M. Farmer, Ariel J. Lang

**Affiliations:** 1 Center for the Study of Healthcare Innovation, Implementation, and Policy (CSHIIP), VA Greater Los Angeles Healthcare System, Los Angeles, CA USA; 2 Long School of Medicine, UT Health Science Center San Antonio, San Antonio, TX USA; 3 David Geffen School of Medicine, University of California Los Angeles, Los Angeles, CA USA; 4 VA San Diego Healthcare System Center of Excellence for Stress and Mental Health, San Diego, CA USA; 5 University of California San Diego, San Diego, CA USA

**Keywords:** Perinatal mental health, Veterans, Collaborative tailoring, Adaptation, Contextual conditions, Qualitative case study

## Abstract

**Background:**

Reach Out, Stay Strong Essentials (ROSE) is an evidence-based intervention for preventing post-partum depression being implemented across U.S. Veterans Health Administration (VA) sites as part of the EMPOWER 2.0 implementation trial comparing Replicating Effective Programs (REP) and Evidence-Based Quality Improvement (EBQI) strategies for improving women’s health care. As both REP and EBQI support adaptation to meet local needs, the EMPOWER 2.0 implementation team and participating sites have collaboratively developed adaptations of ROSE to better serve pregnant veterans. We describe contextual conditions arising during the first three years of implementation, associated adaptations to the intervention and implementation approach, and implications for pragmatic tailoring and diffusion of evidence-based interventions.

**Methods:**

We conducted a qualitative case study that included rapid qualitative analysis of 50 periodic reflections (brief guided discussions with templated notes) completed with EMPOWER 2.0 implementation team members February 2021-February 2024. Contextual conditions were characterized according to domains of the updated Consolidated Framework for Implementation Research (CFIR); adaptations were characterized using the Framework for Reporting Adaptations and Modifications to Evidence-Based Interventions (FRAME) and Framework for Reporting Adaptations and Modifications to Evidence-based Implementation Strategies (FRAME-IS).

**Results:**

Sites reported high demand for ROSE in response to perceived gaps in care for pregnant veterans’ mental health needs. Site-level challenges included the need to locate ROSE within existing services, although the salience of contextual conditions evolved across implementation phases. Notable adaptations included updates to the ROSE intervention (e.g., improving alignment with VA clinical practice guidelines) and adaptations to the implementation approach (e.g., offering training to VA providers beyond the original EMPOWER sites). Although the trial is ongoing, expansion of ROSE training has resulted in a total of 256 VA providers trained across 48 VA facilities nationwide.

**Conclusions:**

In implementing ROSE across a national sample of VA sites, co-produced adaptations emerged to improve feasibility of delivery and increase acceptability of ROSE for pregnant veterans. Implementation of ROSE in EMPOWER 2.0 provides a pragmatic model for supporting rapid iteration and diffusion of adaptations to address perinatal mental health needs within large healthcare systems.

**Trial registration:**

ClinicalTrials.gov: Enhancing Mental and Physical Health of Women Veterans (NCT05050266).

Registration Date: 09/09/2021.

https://clinicaltrials.gov/study/NCT05050266?term=EMPOWER%202.0&rank=1.

## Contributions to the literature

- We report on a qualitative case study of implementing Reach Out, Stay Strong Essentials (ROSE), an evidence-based intervention for preventing perinatal depression, in the U.S. Veterans Health Administration (VA).
- Contextual conditions and emerging adaptations evolved over the course of ROSE implementation, highlighting the value of collaborative strategies for tailoring interventions in response to implementation challenges.
- Collaborative tailoring of ROSE implementation in the EMPOWER 2.0 trial offers a pragmatic model for rapid iteration and diffusion of adaptations across large integrated healthcare systems.

## Background

Perinatal depression describes onset of depressive symptoms during pregnancy or the first 12 months post-partum, and is associated with increased risk of suicidal ideation for mothers and long-term mental and behavioral health problems in children [1–5]. Perinatal depression is common globally, with a meta-analysis estimating pooled prevalence of 24.7% across 8000 studies conducted in 51 countries [6]. In the United States, estimates of perinatal depression prevalence range from 15 to 25%, with evidence of higher rates among women who are rural, Black, or living in disadvantaged neighborhoods [7–10]. Women veterans, who are more likely than male veterans and non-veteran women to be from minoritized racial or ethnic groups [11, 12], may also experience stressors that further increase risk for perinatal depression, including military sexual trauma and posttraumatic stress disorder [13, 14]. Studies have identified depression rates among veterans during pregnancy and postpartum as high as 28–30% [15–17], with associated increases in suicidal ideation [15] and poorer maternal-infant bonding [18].

Access to perinatal mental health treatment is considered a “serious gap” across the U.S [19], and improving perinatal mood disorder management was recently identified as a top implementation research priority [20]. Fortunately, evidence-based interventions for the prevention of perinatal depression are available. The U.S. Preventive Services Task Force recommends counseling interventions such as cognitive behavioral therapy and interpersonal therapy for prevention, and cites the Reach Out, Stay Strong Essentials (ROSE) program as an interpersonal therapy exemplar [21]. Outside of the VA, ROSE has been the subject of five randomized controlled trials, including among women from low-income, rural, and racial/ethnic minority groups, and has been shown to reduce risk of developing postpartum depression by up to 50% [22–26]. ROSE teaches skills to improve communication and bolster social support and can be delivered by non-clinicians, such as health educators, in small outpatient groups, which typically comprise four 90-minute sessions during pregnancy and a follow-up “booster” session post-delivery.

The “Enhancing Mental and Physical Health for Women Veterans through Engagement and Retention” (EMPOWER) 2.0 Quality Enhancement Research Initiative (QUERI) is rolling out ROSE as part of a cluster-randomized hybrid type 3 implementation-effectiveness trial comparing the impact of Replicating Effective Programs (REP) and Evidence-Based Quality Improvement (EBQI) as phased strategies for implementing evidence-based practices for women veterans in Department of Veterans Affairs (VA) health care [27]. We selected REP and EBQI because both strategies integrate multi-level partner engagement and adaptation, and engaged adaptation can improve the appropriateness, reach, and effectiveness of interventions, particularly in new settings or populations [28, 29].

The current article responds to two calls in the literature. The first is for prioritizing implementation research to reduce the maternal health crisis [20, 30]. The second is for better understanding the relationship between contextual conditions, or “features of the circumstances in which an intervention is implemented…that may produce variation in outcomes” [31], and adaptations that emerge over the course of implementation, typically in an effort to improve the fit between intervention and setting [32, 33]. In response, the current manuscript reports on a qualitative case study of contextual conditions impacting the first three years of ROSE implementation within VA, as well as adaptations to the ROSE intervention and EMPOWER implementation approach that have emerged in collaborative, multi-level efforts to better meet the needs of pregnant veterans. We close by considering implications for rapid and pragmatic tailoring and diffusion of interventions to improve perinatal mental health in large healthcare systems like the VA.

## Methods

### EMPOWER 2.0 study overview

The EMPOWER 2.0 QUERI was launched as a hybrid type 3 effectiveness-implementation trial to address disparities in women veterans’ health outcomes [27]. The trial focuses on implementing three virtual evidence-based practices—ROSE; Diabetes Prevention Program (DPP), shown to reduce risk of incident type 2 diabetes [34]; and Telephone Lifestyle Coaching (TLC), which provides supportive coaching for healthy behaviors to reduce cardiovascular risk [35]—across at least 20 VA sites nationally. Sites are randomized to implement using either REP [36, 37] or EBQI [38, 39], both proven effective in VA healthcare settings. REP, a lower-intensity strategy, follows a phased approach that integrates intervention packaging, training and technical assistance, and re-customization in implementation/sustainment phases [36]. The EMPOWER team integrated REP with multi-level partner engagement and complexity theory in an earlier trial [37, 40, 41], and has maintained an engaged REP approach in the current work. EBQI, as a higher-intensity strategy, offers a step-by-step quality improvement method for engaging multi-level providers, staff, and leadership in improvement efforts that include enhanced tailoring, improvement cycles, and external facilitation [38]. Both strategies emphasize collaborative partnership and adaptation for successful implementation [19, 30].

**Fig. 1:**
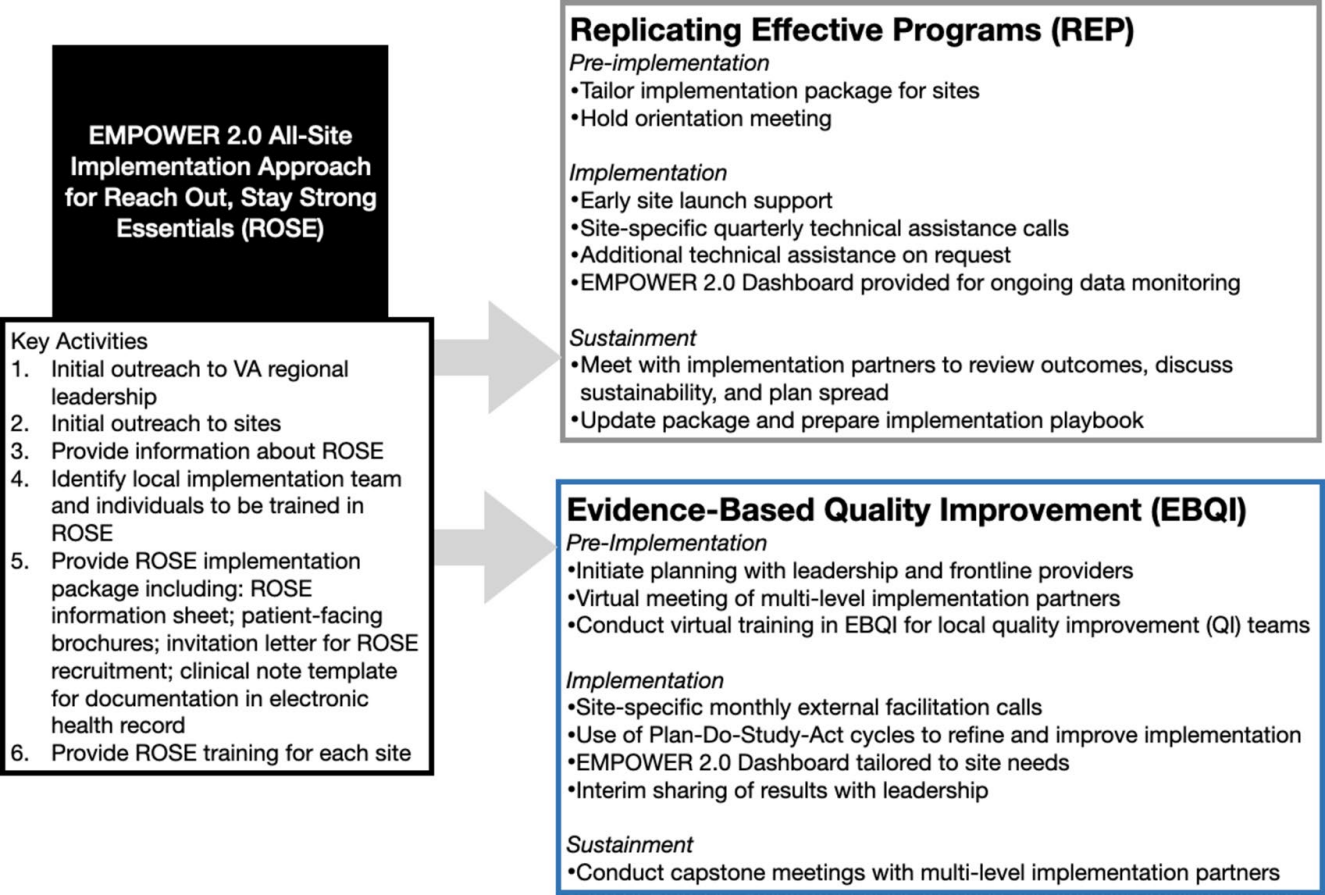
EMPOWER 2.0 All-site implementation approach and implementation activities for sites randomized to REP or EBQI

All EMPOWER 2.0 sites were given the option to implement ROSE, regardless of the implementation strategy to which they were randomized. Sites choosing to deliver ROSE were offered an initial all-site implementation approach (i.e., received by both REP and EBQI sites, see Fig. 1) that included information about the intervention, identifying a local implementation team and individuals to be trained in the ROSE intervention (e.g., potential facilitators for ROSE groups, providers likely to be referring to ROSE), and a ROSE implementation package that included patient-facing brochures, a recruitment letter, and clinical note template to aid in documenting ROSE patient recruitment and encounters in the VA electronic health record. Pre-implementation meetings were held with all sites to review the package and invite feedback on how elements might need to be tailored to suit local needs and context. Participating sites were trained by the ROSE training lead, a clinical psychologist, in one-time virtual sessions. Additional implementation support was provided based on sites’ REP or EBQI assignments, including quarterly technical assistance calls for REP sites and specialized EBQI training and monthly facilitation calls for EBQI sites; these meetings, although structured differently and occurring at different frequency by REP vs. EBQI condition, provided additional opportunities to discuss emergent challenges and adaptations that had been developed or might be needed to improve feasibility and acceptability of ROSE implementation at the site. Because the goal was to compare the pragmatic effectiveness of REP and EBQI strategies for implementing ROSE, no funding or compensation was provided to sites or participants; members of the EMPOWER team, including the training lead and project team, were compensated as part of their existing roles to offer centralized implementation support. The EMPOWER 2.0 trial was deemed to be non-research by the VA Office of Patient Care Services (approval 11/26/19) prior to funding.

### Data collection

To examine contextual conditions and adaptations to the ROSE intervention and the all-site implementation approach in EMPOWER 2.0, we collected longitudinal data between February 2021 and February 2024. We employed a qualitative case study design, which is recommended for bringing together multiple data sources (including quantitative if appropriate) to develop a comprehensive understanding of a specific event or process [42–44].

#### Periodic reflections

The EMPOWER team previously developed periodic reflections as an ethnographically-informed method of guided discussions with key implementation participants to ensure consistent documentation of implementation activities, events, and contextual conditions [45]. Reflections are brief (15–45 min) and recur frequently (every 1–2 months) throughout implementation, minimizing documentation burden for busy implementation teams. By focusing data collection efforts on individuals and teams central to implementation, reflections rely on a purposeful, information-power-based sampling approach [46]. As part of the mixed-method evaluation for the EMPOWER 2.0 trial [27], we conducted periodic reflections with EMPOWER team members actively supporting implementation and/or engaging with partners at national, regional, or site levels. Participating roles for reflections were selected as part of the initial study design to ensure comprehensive overview of implementation activities, including nine individuals: the ROSE implementation lead, ROSE training lead, and seven project directors providing REP or EBQI implementation support to sites. All reflections were conducted virtually or via telephone by a PhD-level medical anthropologist (EPF) using a semi-structured discussion guide (previously published [27]); specific prompts invite discussion of recent implementation activities, challenges and areas of progress, changes to the intervention or implementation approach, recent changes in the implementation environment, and planned next steps. Content of reflections was documented using near-verbatim notes or Microsoft Teams’ recording and transcription feature.

#### ROSE training and site-level adoption

EMPOWER 2.0 was the first VA-based study to use the Stages of Implementation Completion (SIC) webtool for documenting key milestones (e.g., completing ROSE training) at the site level [47, 48]. We used SIC data to characterize the number, VA healthcare region (i.e., Veterans Integrated Service Network [VISN]), and geographic spread of EMPOWER sites that completed the required ROSE training. We also drew upon EMPOWER 2.0 training attendance logs to identify the number of training attendees per site during this period.

### Analysis

We conducted rapid qualitative analysis of all 50 periodic reflections completed with nine members of the ROSE implementation team during this three-year period, in accordance with recommended best practices for ensuring rigor in rapid analyses [49]. All text relevant to ROSE implementation was first extracted into a single file in Microsoft Word. Extracted text was then summarized into a templated summary table, organized by reflection date (rows), that distilled brief bullet points and illustrative quotes relevant to each of the following topic domains (columns): barriers and challenges; facilitators and supports; and adaptations to the ROSE intervention or implementation approach. Summary rows were combined into a matrix collating the content of domains by implementation phase (e.g., engagement, pre-implementation, implementation, sustainment). Within each phase, summarized data relating to barriers/challenges and facilitators/supports were then organized by key overarching domains of the updated Consolidated Framework for Implementation Research (CFIR 2.0) (outer setting, inner setting, innovation, implementation process), and further by subconstruct (e.g., innovation adaptability) where appropriate [50]. Summarized data related to adaptations were similarly collated into adaptations to the ROSE intervention, which were characterized according to the Framework for Reporting Adaptations and Modifications to Evidence-based interventions (FRAME) [51], and adaptations to the ROSE implementation approach, which were characterized according to the Framework for Reporting Adaptations and Modifications to Evidence-based Implementation Strategies (FRAME-IS) [52]. Although some studies operationalize “adaptations” as referring only to planned or proactive changes made prior to launching implementation of an intervention in a new context [53], we conceptualize such changes in line with the more recent definition proposed by the FRAME authors of adaptations as “thoughtful or deliberate modifications made to the intervention or implementation strategies, with the goal of improving their fit with a given context,” and which they note may be proactive or reactive [54]. Because comparison of REP vs. EBQI strategies is a primary aim of the EMPOWER 2.0 trial, we adopted multiple safeguards to ensure fidelity to REP and EBQI conditions [55] and adaptations were only made to the implementation approach received by all sites. Preliminary analysis was conducted by three authors (EPF, AH, AL) and presented back to the full EMPOWER 2.0 team, including reflection participants, to allow for member checking and ensure accuracy and completeness of findings [56, 57]. Team members provided substantive feedback that aided in verifying contextual conditions and adaptations emerging during this period.

## Results

Over the course of initial ROSE rollout within the EMPOWER 2.0 trial, we observed evolution in the contextual conditions (e.g., outer setting, inner setting) that emerged as most salient across implementation phases, as well as in the associated adaptations made to the ROSE intervention and all-site implementation approach. Below we review contextual conditions and adaptations by implementation phase and emerging trends in the nature and ownership of adaptations across phases. We close with an overview of preliminary outcomes in ROSE training across sites.

### Engagement

**Fig. 2:**
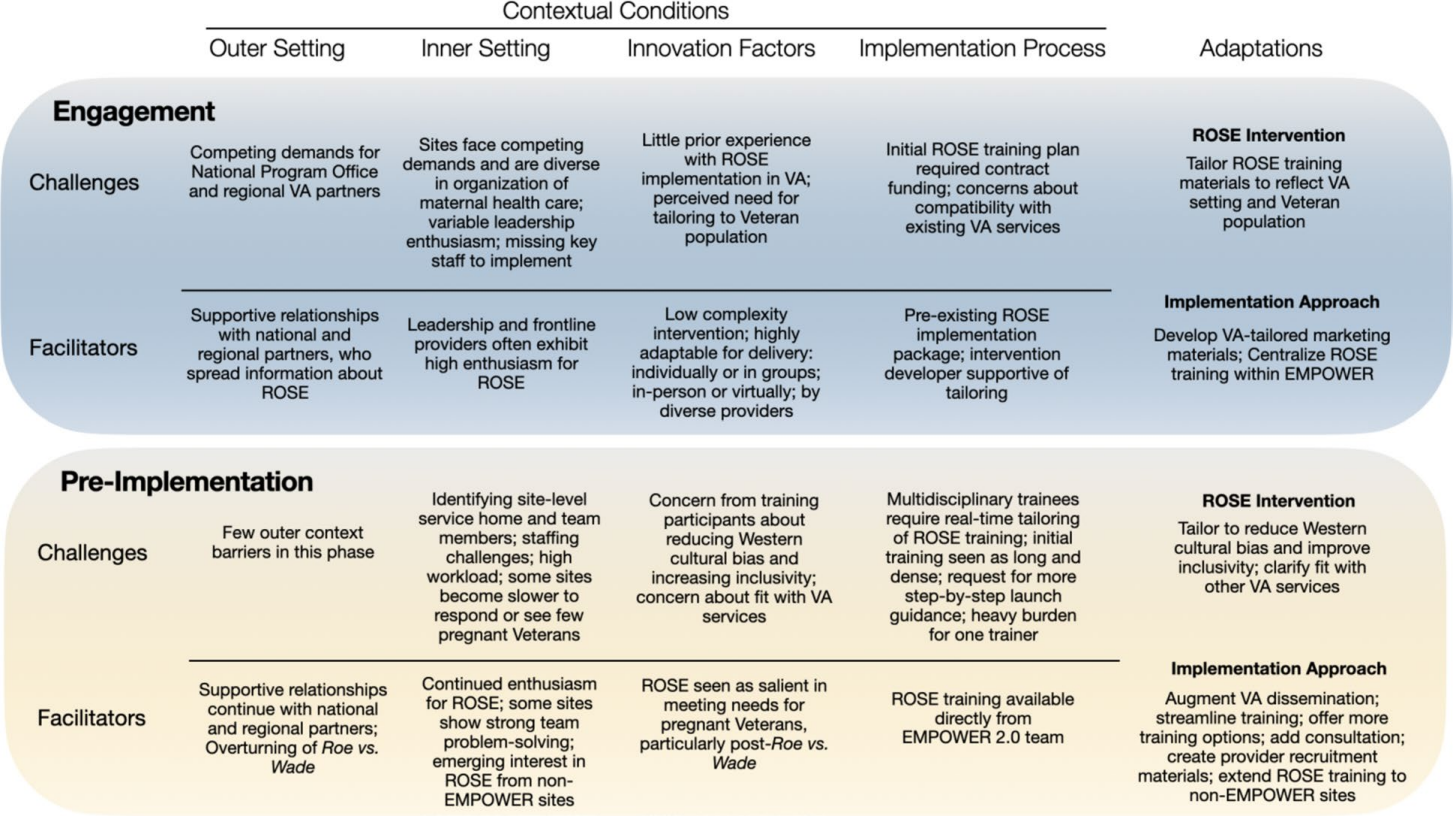
Contextual conditions and adaptations in engagement and pre-implementation phases of ROSE implementation: February 2021 – February 2024

Initial EMPOWER 2.0 activities involved connecting with partnering VA program offices, including the Office of Women’s Health and the Office of Mental Health’s Women’s Mental Health Program, regional VISN-level partners, and individual sites to gauge interest in ROSE. Early outer setting challenges (see Fig. 2) included competing demands at the national and regional levels and unfilled women’s health leadership positions in some VISNs. At the site (inner setting) level, it was sometimes difficult to identify the most compatible organizational home and/or provider team to champion ROSE, for several reasons. First, ROSE can be delivered by diverse professions (e.g., psychologists, social workers, nurses), leaving no single clear role to lead implementation. Second, VA does not offer obstetric or pediatric services, and perinatal mental health care occurs at the intersection of maternity care and mental health provision, creating no obvious home for the program. Moreover, VA medical centers vary in how they organize women’s health care, with some co-locating women’s health, mental health, and primary care services, and some offering these in distinct spaces or locations. Some sites were already invested in other maternity care services or missing key staff to implement the program. Related to the innovation itself, ROSE had previously been implemented in only a few VA sites, offering little precedent for how to launch in the VA context. Implementation process-related challenges included ensuring compatibility with VA organizational structures and existing services.

Despite these early challenges, contextual conditions largely supported momentum in engaging partners for ROSE implementation. The EMPOWER 2.0 team had a strong existing relationship with national program partners, who were supportive of implementing ROSE in VA. Some VISN-level regional partners offered more active buy-in and involvement than anticipated, for example, by marketing ROSE to site-level leadership. Moreover, VISN-level partners began sharing ROSE information with sites that were not enrolled in the EMPOWER trial (hereafter referred to as non-EMPOWER sites), sparking broader interest. At the site level, enthusiasm for ROSE was often high from both leadership and frontline providers. The ROSE implementation lead noted of one site, “The Women’s Health Care Coordinator said she’s very excited about ROSE, that she has an action plan and she can’t wait to do it.”

Several features of the ROSE intervention were felt to facilitate implementation. It is a low-complexity intervention and highly adaptable to local needs and resources, allowing delivery in individual or group sessions and virtually as well as in-person. Early engagement was aided by pre-existing, publicly available ROSE training and intervention materials in digital format, and ROSE intervention developer Dr. Caron Zlotnick was open to adaptation as needed.

Adaptations to the ROSE intervention during the engagement period focused on tailoring training and educational materials to address women veterans’ needs and the VA environment; for instance, by removing descriptions of ROSE as a psychotherapy, which would limit the professions allowed to deliver the intervention within VA. Adaptations to the implementation approach were similarly focused on tailoring for VA and included developing site-facing informational materials to address key questions, such as who at the site would need to be involved.

### Pre-implementation

As the engagement period transitioned into pre-implementation planning, challenges in the inner setting intensified as sites navigated how ROSE would integrate within local VA structures for women’s health, primary care, and mental health. The ROSE implementation lead reported, “There’s a lot of fact-finding about where this is going to happen,” and found that sites sometimes struggled to identify team members to take ownership and “be the point person” at the local level. Sites frequently reported feeling overburdened with existing responsibilities, or were understaffed or lacking personnel in key positions, such as the Maternity Care Coordinator, whose role was critical to identifying veterans eligible for referral to ROSE. Some smaller sites, particularly in rural areas, questioned whether there were enough pregnant veterans at their sites to conduct ROSE in group sessions as planned. Some sites, despite initial enthusiasm, became slower to respond to the EMPOWER team’s efforts to schedule meetings, trainings, or implementation support calls.

Also during this period, as the first EMPOWER sites were trained to deliver ROSE, questions arose around tailoring the ROSE intervention to meet VA patient needs. For example, concerns were raised regarding Western bias in how key concepts were discussed, such as assertiveness, which the ROSE intervention highlights as a skill for negotiating social support. The ROSE training lead noted that assertiveness is: “not necessarily a characteristic that is valued outside of Western culture, particularly for women. And so that may not resonate and may be met with more resistance. You may run into acculturation stress.” Relevant to implementation process, attendees at ROSE training sessions were from a broad array of professional backgrounds and brought variable training and experience to ROSE delivery, requiring real-time adjustments by the ROSE trainer. Early feedback on the ROSE training indicated that “it was really good but a lot of information and material” (ROSE implementation lead). Some sites requested more step-by-step guidance on how to implement ROSE in their local settings: “what stop code do we use, what procedure code do we use, what clinic do you set up… stuff like that” (ROSE training lead). One site also requested continuing education credits for the ROSE training, which the EMPOWER team was able to introduce for all trainees with support from the National VA Women’s Mental Health Program. The need to train multiple sites during this period placed heavy demand on the single trainer.

Amid the challenges of this period, continued interest in ROSE from national and regional VA partners remained an outer-setting facilitator. In 2022, the U.S. Supreme Court overturned the *Roe vs. Wade* decision that had for decades protected the right to abortion, and several states enacted legislation prohibiting or reducing access to abortion. At the local, inner-setting level, many sites remained highly motivated to implement ROSE, and sites in states affected by the new legislation reported viewing the ROSE intervention as even more valuable in the post-Roe environment: “They said they had three or four women…who wanted to terminate their pregnancies and couldn’t because of the states that they live in, and so they’re dealing with the mental health kind of fallout from that, and that they would really benefit from a program like this” (ROSE implementation lead). Unexpectedly, a growing number of non-EMPOWER sites began reaching out to the EMPOWER team to request ROSE training, signaling broader interest in making ROSE available to veterans.

The pace of adaptations to the ROSE intervention and implementation approach increased as preparations for implementation progressed. Responding to concerns about Western cultural bias and inclusivity, the ROSE training lead incorporated greater acknowledgment of bias into ROSE training materials. She also emphasized listening to the individual veterans’ experiences and family and cultural context and adapting the intervention accordingly. As she described, “That’s really the core of ROSE: listen to the woman, don’t make assumptions.” Moreover, because ROSE had originally been delivered in maternity rather than mental healthcare settings, it became necessary to augment the training with further guidance on ROSE’s compatibility with other VA care services; for example, by clarifying that pregnant veterans with depression could participate in ROSE in addition to medication or individual therapy, provided it did not interfere with their ability to engage fully in other interventions or services.

The greatest number of adaptations at this time, however, were made to the implementation approach. National partners encouraged broader dissemination by inviting ROSE implementation and training leads to present on VA women’s health-focused practice calls. Virtual ROSE training was made more accessible by streamlining the training to reduce its length, offering grouped training sessions across sites (REP sites and EBQI sites were trained separately to avoid cross-arm contamination), and offering on-demand consultation with the ROSE training lead. One particularly active site, struggling to identify local providers to deliver ROSE, developed a set of materials to garner provider interest and supervisor support for dedicating time to ROSE groups, which included information summarizing requirements for involvement (i.e., expected hours/week, number of meetings to attend). In perhaps the most significant adaptation, the EMPOWER 2.0 team decided to offer ROSE training to non-EMPOWER sites, in response to the outpouring of interest from across VA sites and regions. As these sites fell outside of the EMPOWER 2.0 recruitment and randomization strategy, they were not included in the ongoing EMPOWER 2.0 trial or exposed to REP or EBQI implementation strategies. Non-EMPOWER sites were, however, offered the option of on-demand consultation and asked to use the same ROSE clinical notes template used at EMPOWER sites, to facilitate evaluation of ROSE implementation and sustainment.

### Implementation

**Fig. 3:**
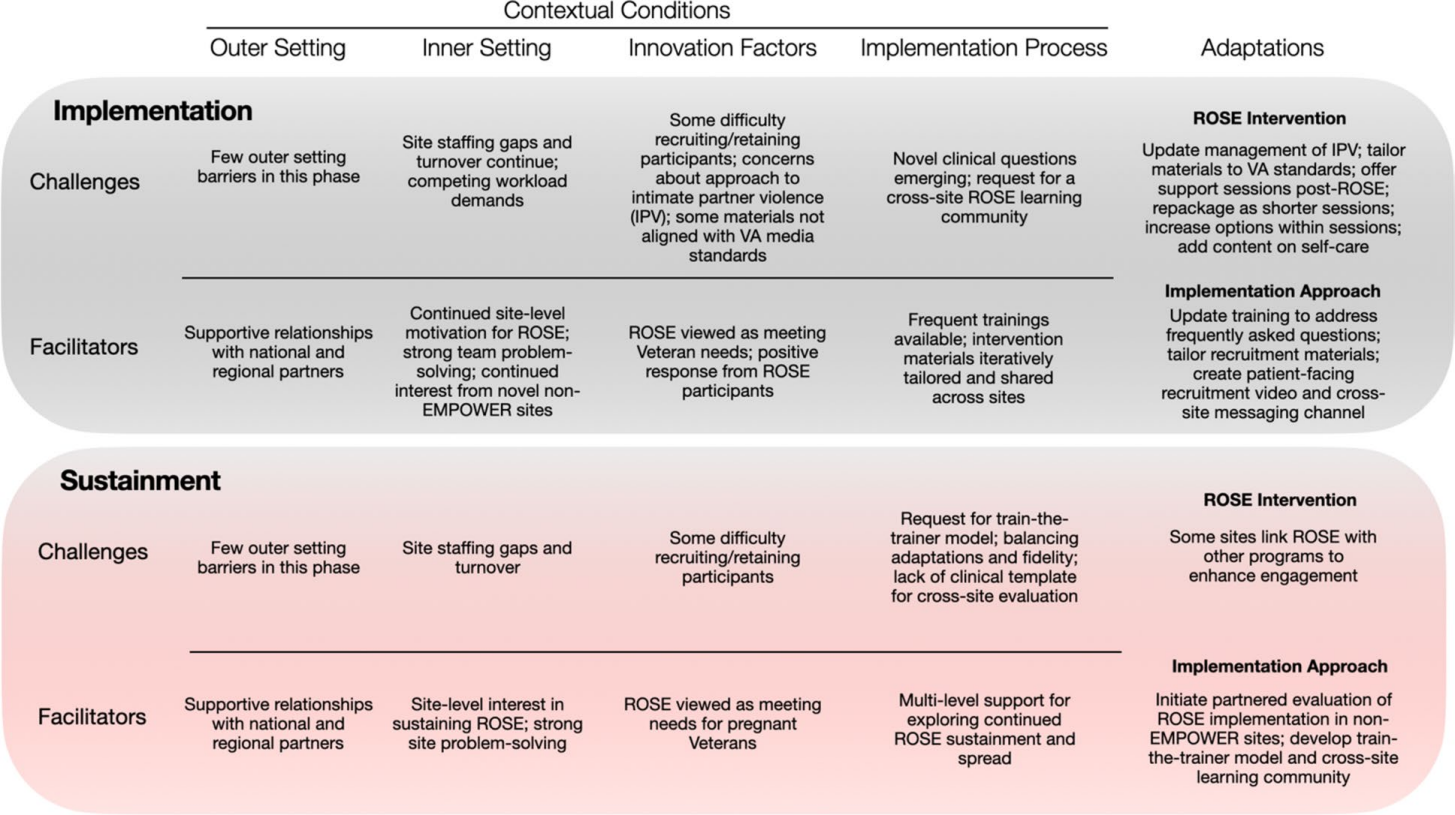
Contextual Conditions and Adaptations in the Implementation and Sustainment Phases of ROSE implementation: February 2021 – February 2024

By the time sites had launched ROSE, as indicated by the first recruitment of pregnant patients, they had often worked past the inner-setting challenges of the pre-implementation period, identifying how to integrate ROSE within local women’s health services using the personnel time and expertise available (see Fig. 3). Some challenges related to staffing turnover and competing workload demands lingered, although these were now more relevant to ensuring continuity of ROSE delivery: “They lost their Maternity Care Coordinator so…they don’t have a stable person who can help with pulling the sample” (Project Director A). However, in this new phase, some sites began to report difficulty with recruiting pregnant veterans to participate in ROSE sessions or found that veterans came to a few sessions but did not complete the program. Several sites expressed concerns regarding how intimate partner violence (IPV) was discussed and managed in ROSE, finding the language “oversimplified” and poorly aligned with VA practice standards. ROSE’s original patient-facing materials for recruitment and education were similarly out of alignment with VA’s medical media standards, which impeded one site’s ability to print and disseminate materials. As sites stood up ROSE delivery, they frequently raised new clinical questions (e.g., can pregnant and postpartum veterans participate in the same group session?).

The outer setting remained positive for ROSE during this phase, with supportive national and regional partners and a continued sense of ROSE’s value in meeting veteran needs in the post-*Roe* era. Implementing sites demonstrated motivation and ownership for ROSE; for example, one site found a novel way to recruit pregnant veterans using a VA-based Coordinated Care tracking system, while another developed a workflow to ensure recruitment could continue when the Maternity Care Coordinator position was unfilled. Meanwhile, novel non-EMPOWER sites continued to request ROSE training. Relevant to the innovation itself, sites running ROSE groups often reported that veteran participants found the intervention to be of value: “[Site] is ecstatic about ROSE, because they’re such a rural site, it’s a lot of women veterans together who otherwise wouldn’t have [the opportunity] to talk about their experience in the military and their relationships and interests” (Project Director B).

Adaptations to both the intervention and the implementation approach continued at a rapid pace during implementation. Concerns about the handling of IPV in the original ROSE led to language updates throughout the training and guidance on discussing healthy relationships in ROSE sessions. A second round of tailoring was led by one of the EMPOWER sites, who updated ROSE to align IPV-related content with VA clinical practice guidelines (e.g., adding guidance specific to safety planning). Another site revised ROSE materials to meet VA medical media standards. In both cases, revised materials were then shared with the EMPOWER team, who – because the adaptations improved compatibility with broader VA guidelines – elected to integrate the changes and disseminate the updated materials to other sites. Additional adaptations arose at some sites reflecting ROSE participants’ desire to continue meeting after the intervention was over (e.g., in a continuing support group), or, alternatively, to improve recruitment and retention by spreading session content across shorter sessions (i.e., six 60-minute sessions rather than four 90-minute sessions). The training lead also began to introduce options for relaxation exercises included as part of ROSE, to make the intervention more compelling: “We’ve always said the relaxation exercise – it’s the concept not the exercise that matters – so I gave them a bunch of other potential tools like meditations and alternative relaxation strategies.” Adaptations to the implementation approach included updating the training to address frequently asked questions, while sites tailored patient recruitment materials to reflect local information and services, created a patient-facing recruitment video, and started a cross-site ROSE messaging channel on Microsoft Teams to support information-sharing and problem-solving.

### Sustainment

Sites that achieved routine ROSE implementation reported fewer challenges as they evolved toward sustainment, although staffing gaps and turnover remained a challenge, particularly when trained ROSE providers were lost or reassigned to other duties. Difficulty with recruiting and retaining participants continued in some sites, raising questions about whether all pregnant veterans are equally likely to benefit from ROSE, or whether the program may need additional tailoring to serve women who are older, busy with work and/or caregiving responsibilities, and/or with greater mental health needs than those in the original non-VA trials. Sites wanting to sustain the program requested a train-the-trainer model to allow local leads to provide training as staff left or joined the team. As sites envisioned a future for ROSE, they frequently considered additional adaptations for delivery, raising questions around balancing adaptation of and fidelity to ROSE; Project Director B noted of one site, “They’re talking about maintaining, but they also have a lot of their own ideas for what they want ROSE to look like going forward.”

Facilitators during the sustainment phase continued to include strong support from national and regional partners, as well as interest in sustaining ROSE and strong implementation teams at the site level.

Adaptations to the innovation emerging during this phase reflected sites’ local sensemaking around how best to engage and serve their pregnant veterans, and primarily took the form of combining ROSE with other local programs. One site integrated ROSE with an existing program of monthly baby showers, while another included ROSE as part of a comprehensive package of maternity care services that also included yoga and nutrition classes. The EMPOWER team’s adaptations to the implementation approach in this phase began to focus on future planning. These included partnering with the VA Office of Women’s Health to conduct rapid evaluation of ROSE implementation in non-EMPOWER sites and developing a train-the-trainer model to support sustainment and continued spread.

### VA-Tailored ROSE: progress to date

**Fig. 4:**
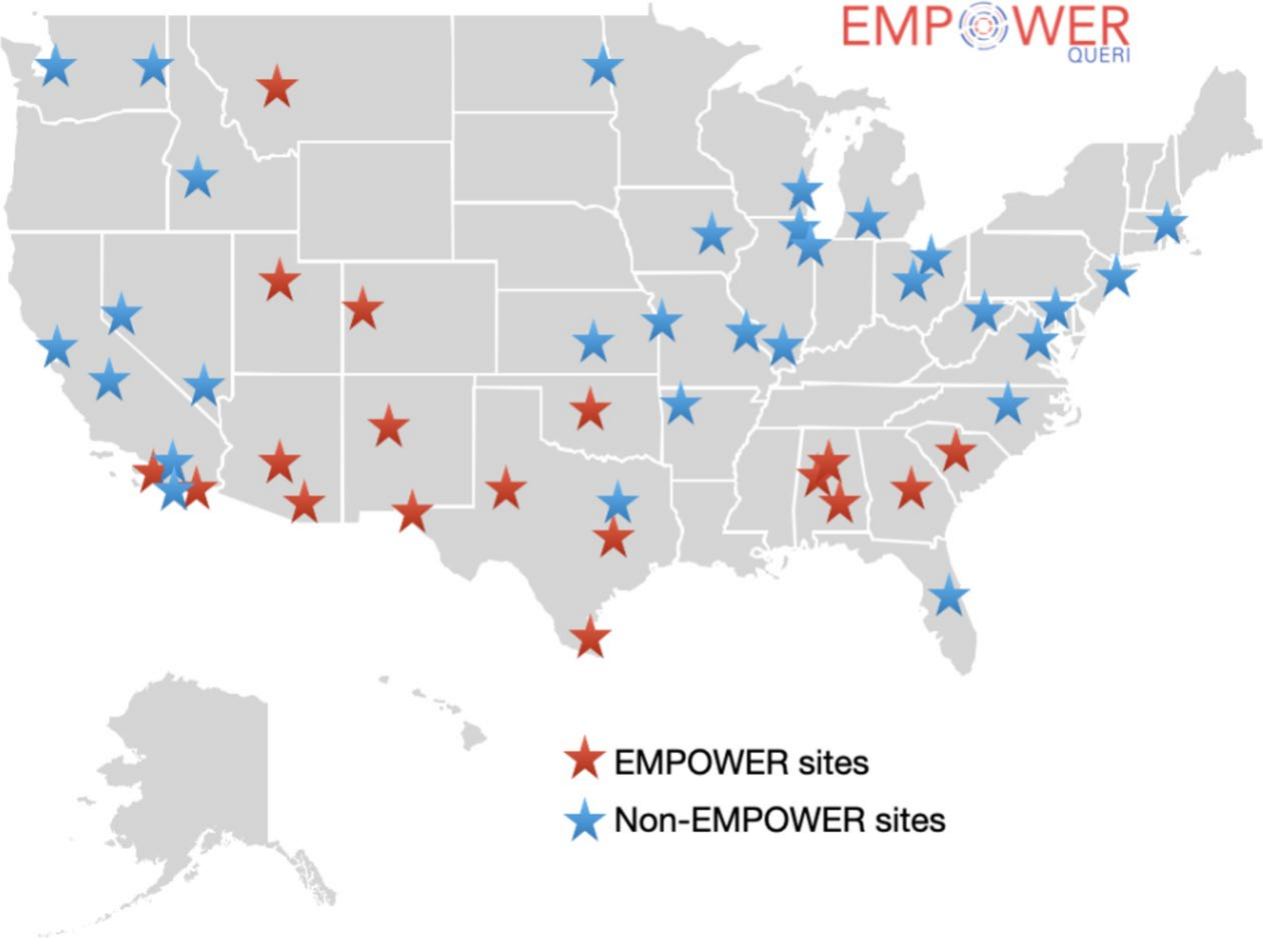
VA Medical Center Facilities with Providers Trained in ROSE: February 2021-February 2024

Although the EMPOWER 2.0 trial will continue until September 2025, considerable progress was made in the implementation and spread of ROSE for pregnant veterans during this period (Fig. 4). As of February 29, 2024, 136 VA providers across 18 EMPOWER sites in four VISNs had been trained in ROSE delivery, and 111 pregnant Veterans had participated in at least one ROSE session at an EMPOWER site. Four of the 18 sites had already achieved implementation competence (defined for this study as meeting a program enrollment goal of 10 pregnant Veterans [27]). Reflecting the adaptation of offering ROSE training to non-EMPOWER sites, an additional 124 VA providers had been trained across 30 non-EMPOWER sites in 14 VISNs (two EMPOWER-engaged VISNs plus 12 novel VISNs).

### Nature and ownership of adaptations across phases

Over the four implementation phases, adaptations to the ROSE intervention (see Table 1) generally emerged in response to novel information gleaned from conducting trainings, collaborating with site-level implementation teams, and teams’ own implementation challenges. Adaptations during the engagement phase were made by the central EMPOWER ROSE team, while the pre-implementation phase saw increasing collaboration between the EMPOWER team and site-level implementation teams, and later phases saw active ownership of adaptations at the site level. Early-phase ROSE adaptations focused on tailoring to improve acceptability and appropriateness for the VA context, while changes during the implementation and sustainment phases frequently involved adding elements to improve intervention fit and increase effectiveness for women veterans.

**Table 1:**
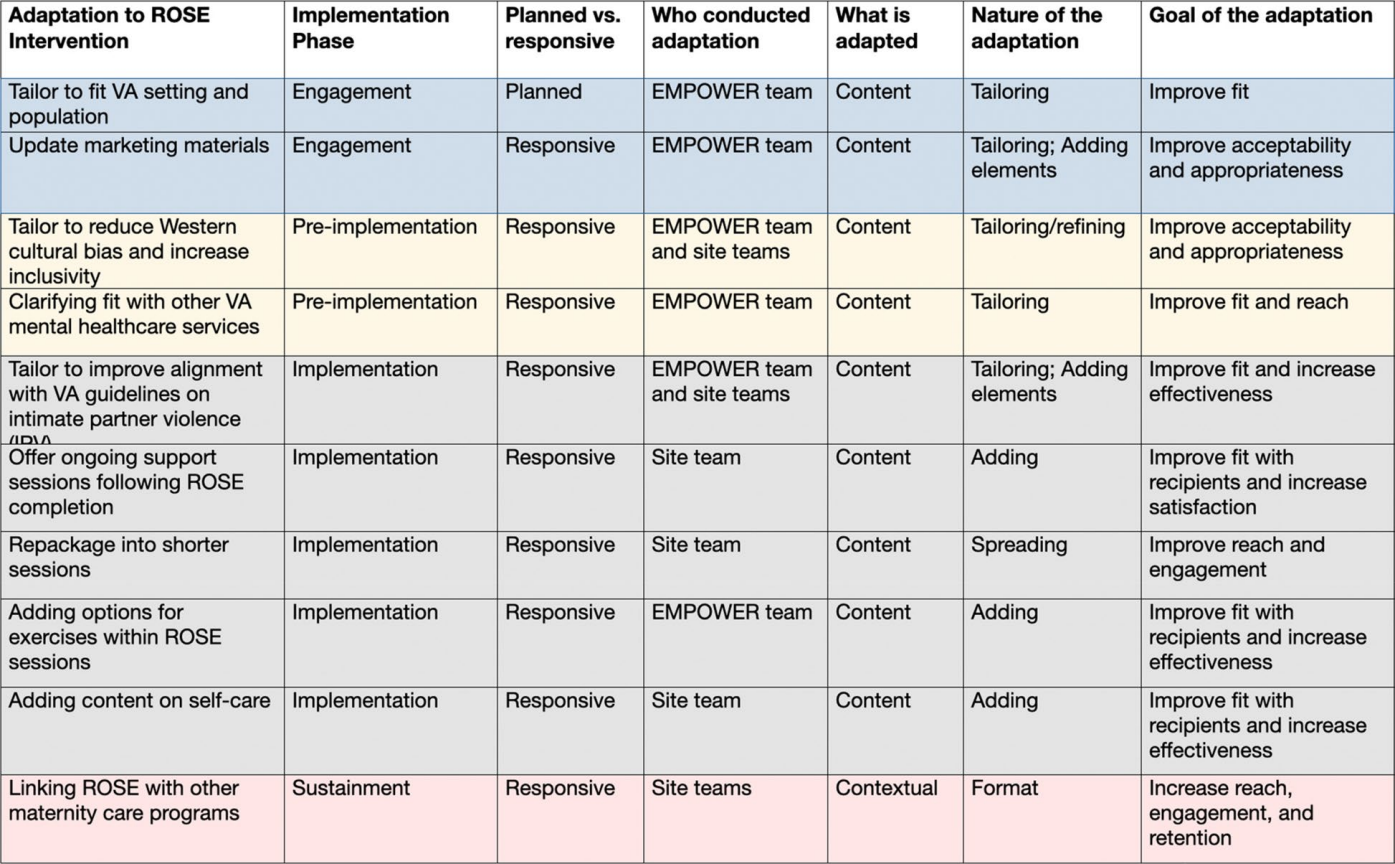
Adaptations to the ROSE intervention by FRAME [51] domains: February 2021-February 2024

Over the four implementation phases, adaptations to the EMPOWER 2.0 all-site ROSE implementation approach (see Table 2) were occasionally planned but more frequently responsive to continuous learning in dialogue with sites. Like intervention adaptations, adjustments to the implementation approach in the engagement and pre-implementation phases tended to be led by the EMPOWER team, often in response to feedback from site-level partners, with increasing involvement and leadership from sites as they entered implementation and began planning for sustainment. Most adaptations to the implementation approach initially took the form of tailoring and integrating strategies to increase ROSE’s acceptability and adoption within VA settings, shifting toward adding elements to increase reach and effectiveness in later stages, as sites responded to emerging challenges in recruiting and retaining pregnant veterans for ROSE sessions. In the sustainment phase, adaptations to the implementation approach reflected collaboration with site-level and national partners, specifically in launching an evaluation of ROSE implementation in non-EMPOWER sites. Adaptations under development include formalizing a train-the-trainer model to ensure continuous workforce capacity for ROSE delivery and developing a learning collaborative to support sites in implementation and continuous improvement of ROSE.

**Table 2:**
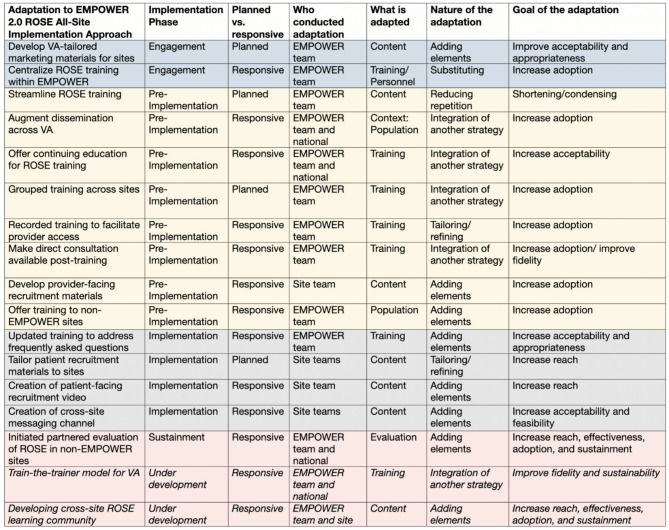
Adaptations to the EMPOWER 2.0 ROSE All-Site implementation approach by FRAME-IS [52] domains: February 2021-February 2024

## Discussion

Over this three-year case study, challenges and facilitators to ROSE implementation in the VA prompted iterative adaptations to the ROSE intervention and the EMPOWER 2.0 team’s implementation approach. These adaptations occurred in the context of multi-level collaborations between EMPOWER’s implementation team, VISN and national leaders, and sites implementing ROSE. We found that the challenges and facilitators most salient to implementation varied across engagement, pre-implementation, implementation, and sustainment phases, as did the nature and ownership of associated adaptations during these periods. In this context of collaboration between EMPOWER’s implementation team and the sites implementing ROSE, we have tailored ROSE and our implementation approach to better meet the needs of VA’s pregnant veterans and organizational structures for perinatal health care. Where adaptations have occurred at the site level, e.g., in the form of VA-tailored patient-facing materials, these were often made available as “menu options” for other sites, facilitating rapid spread and diffusion. Implementation of ROSE in EMPOWER 2.0 thus provides a pragmatic model for timely iteration and diffusion of tailored interventions in integrated healthcare systems.

Although ROSE has a strong clinical evidence base demonstrating its effectiveness in preventing postpartum depression, the findings reported here also illustrate the utility of co-production strategies for tailoring evidence-based interventions to ensure compatibility in new organizational environments and patient populations. VA-based women’s health care for veterans is delivered via one of several distinct models, i.e., by a women’s health primary care provider within a primary care clinic, in a separate-but-shared space within a larger primary care clinic, or in a dedicated women’s health center providing comprehensive care [58]. Moreover, because VA does not directly provide obstetric or pediatric services, care for pregnant veterans may be delivered by a variety of providers, and perinatal mental health care may variably be seen as “belonging to” primary care, women’s health, or mental health. Moniz et al. [59] have argued that maternity care represents one of the more complex settings for implementation due to the need for coordination across professions and organizational boundaries, and that is certainly true for perinatal mental health care in the VA. A recent systematic review of barriers and facilitators to implementing perinatal mental health care concluded that this complexity amplifies the need for implementation approaches that draw upon the co-production of services, multi-level coalition-building, and learning collaboratives [60], all of which emerged organically in this case example.

Although foundational implementation science theories like those underlying the Exploration, Preparation, Implementation, and Sustainment (EPIS) framework [61] might well predict that certain contextual conditions will have greater or lesser salience during different phases of implementation, and may thus be associated with differing adaptations occurring during those phases, few studies have explored this phenomenon longitudinally. McCreight et al. [62] conducted in-depth adaptation tracking across two sites implementing a VA Advanced Care Coordination program and found, similar to our results, that the periods of pre-implementation and implementation were among the most active for adaptations. Although EMPOWER 2.0 is ongoing, the emergence of adaptations in later stages of implementation, including among sites attempting sustainment, underscores the importance of continued monitoring, documentation, and evaluation of adaptations in scale-up and spread [63]. Organizational diversity is the norm rather than the exception – it is a common saying within VA that “if you’ve seen one VA, you’ve seen one VA” – and that diversity becomes increasingly impactful as an intervention moves into new care systems or is used with new populations [28, 64]. In the wake of the COVID-19 pandemic, it may be self-evident that historical events and policy are significant elements of the outer setting, but this, too, was illustrated in these findings, as the overturning of *Roe vs. Wade* was cited by several sites as providing added impetus to implement ROSE.

Finally, in line with other recent studies addressing this issue [63], the ROSE case study illustrates how adaptation and refinement of evidence-based interventions and implementation strategies can occur in the context of a rigorous implementation trial. It is our hope that the methods described here provide a pragmatic model for facilitating and documenting longitudinal adaptation even within larger-scale implementation efforts utilizing hybrid or other robust trial approaches.

### Limitations

We acknowledge several limitations of this analysis. Reliance on qualitative data gathered through periodic reflections conducted with the implementation team, while providing valuable descriptive and observational data, may underplay contextual factors relevant to the characteristics of individuals delivering or receiving the intervention. We hope to further investigate individual-level factors of relevance to ROSE implementation in future work, including through semi-structured interviews with VA providers and pregnant veterans. In addition, the information on adaptations provided by periodic reflections may not be comprehensive, as this method offers less precise detail than do comparable methods for adaptation tracking, such as tracking logs [62, 65]. However, reflections have the benefit of being lower burden for active study teams and may therefore offer the tradeoff of more consistent completion. Moreover, implementation team members may not always appreciate activities as “adaptations” amid the day-to-day bustle of intervention rollout. Following a dedicated process for identifying, describing, and verifying adaptations in regularly gathered reflections data, like that described here, may allow for enhanced recognition of adaptations by harnessing both consistent data capture and team discussion. The rigor and trustworthiness of this case study have been increased by: high information power among participants, who could report both on their own experience of conducting and observing implementation as well as their direct interactions with sites [46]; use of an ethnographically informed method for gathering data throughout implementation, rather than at limited timepoints, thus decreasing the likelihood of recall bias [45]; and use of multiple strategies for verifying qualitative data, including confirming internal coherence between the research question and methods and iterative member-checking of findings [56, 57, 66]. Given data currently available, we are not able to speak to the impact of specific adaptations in improving adoption, reach, or other outcomes [67], but look forward to examining these associations in future analyses. For example, we anticipate conducting mixed-method analyses examining whether a site’s exposure to specific adaptations was associated with their progression to ROSE implementation or sustainment or with veteran engagement and retention in ROSE sessions at that site. We are also unable to thoroughly assess the extent to which adaptations impacted our strategies. Future implementation would benefit from carefully examining and documenting this interplay.

## Conclusions

Although the EMPOWER 2.0 trial is ongoing, collaborative tailoring of the ROSE intervention and implementation approach across sites has resulted in an evidence-based intervention for the prevention of perinatal depression that is compatible with the VA care environment and shows promising signs of achieving sustainment and wider spread. In the next phase of work, we aim to continue working directly with VA sites and pregnant veterans to further refine the model of care and ensure its utility and effectiveness for all pregnant veterans.

## Data Availability

The datasets generated and/or analysed during the current study are not publicly available as participants have not provided consent for sharing; de-identified portions may be available from the corresponding author on reasonable request.

## Abbreviations

DPP: Diabetes Prevention Program
EBP: Evidence-Based Practice
EBQI: Evidence-Based Quality Improvement
EMPOWER: Enhancing Mental and Physical Health of Women Veterans through Engagement and Retention
QUERI: Quality Enhancement and Research Initiative
REP: Replicating Effective Programs
ROSE: Reach Out, Stay Strong Essentials
TLC: Telephone Lifestyle Coaching
VA: Veterans Affairs
VAMC: VA Medical Center
VISN: Veterans Integrated Service Network
